# Supplementary material for: Comparative Modelling of Organic Anion Transporting Polypeptides: Structural Insights and Comparison of Binding Modes
Source: Molecules. 2022 Dec 3;27(23):8531. doi: 10.3390/molecules27238531 (PMC9738416; doi:10.3390/molecules27238531)
Supplement: Supplementary file 1 [file molecules-27-08531-s001.zip › molecules-2026282-supplementary.pdf]

Supporting Information

Comparative Modelling of Organic Anion Transporting Polypeptides (OATPs): Structural Insights and Comparison of Binding Modes

Arun Kumar Tonduru [1], Santosh Kumar Adla [1,2], Kristiina M. Huttunen [1], Thales Kronenberger [1,3*] and Antti Poso [1,3*]

1. School of Pharmacy, Faculty of Health Sciences, University of Eastern Finland, P.O. Box 1627, 70211 Kuopio, Finland
2. Institute of Organic Chemistry and Biochemistry (IOCB), Czech Academy of Sciences, Flemingovo Namesti 542/2, 160 00 Prague, Czech Republic
3. Department of Internal Medicine VIII, University Hospital Tuebingen, Otfried-Müller-Strasse 14, Tuebingen DE 72076, Germany. Department of Pharmaceutical and Medicinal Chemistry, Institute of Pharmaceutical Sciences, Eberhard-Karls-Universität, Tuebingen, Auf der Morgenstelle 8, 72076 Tuebingen, Germany. Cluster of Excellence iFIT (EXC 2180) "Image-Guided and Functionally Instructed Tumor Therapies", University of Tübingen, 72076 Tuebingen, Germany. Tuebingen Center for Academic Drug Discovery & Development (TüCAD2), 72076 Tuebingen, Germany

*Author to whom correspondence should be addressed. T.K, A.P.

List of Tables:

Table S1: Model validation report from Ramachandran plots

Table S2: SiteMap scores for the binding site regions of OATPs. SiteMap prediction for the potential binding sites in all OATPs. The SiteScore is >0.8 for all the OATPs which depicts the druggability of the sites. The table also includes the metrics calculated by the SiteMap Volume, size, Hydrophobic, hydrophilic and donor/acceptor sites. SiteMap scores for the binding site regions of OATPs.

Table S3: RMSD (Transmembrane helices) calculated by aligning alpha fold models and our OATP models.

List of Figures:

Figure S1: OATP1A2 alignment with 6E9N and 1TGS

Figure S2: OATP1B1 alignment with 6E9N and 1TGS

Figure S3: OATP1B3 alignment with 6E9N and 1TGS

Figure S4: OATP1C1 alignment with 6E9N and 1TGS

Figure S5: OATP2A1 alignment with 6E9N and 1TGS

Figure S6: OATP2B1 alignment with 6E9N and 1TGS

Figure S7: OATP3A1 alignment with 6E9N and 1TGS

Figure S8: OATP4A1 alignment with 6E9N and 1TGS

Figure S9: OATP4A1 alignment with 6E9N and 1TGS

Figure S10: OATP5A1 alignment with 6E9N and 1TGS

Figure S11: OATP6A1 alignment with 6E9N and 1TGS

Figure S12: Distances along the molecular dynamics simulations of OATPs. A) Distance between Ser/Thr on TM2 vs Glu on TM1 observed along the simulations. B) Distance between Ser/Thr on TM2 vs Lys on TM7.

Figure S13: Identity and similarity matrices of binding sites of OATPs: A) Identity matrix of binding sites of OATPs. B) Similarity matrix of binding sites of OATPs.

Figure S14: Classification of OATPs based on the interactions observed in the molecular dynamics simulations.

Figure S15: Frequency of protein-ligand interactions along the molecular dynamics simulation trajectories are shown in bubble graphs, the residues are named according to OATP1A2, and the corresponding aligned residues could be referred from Figure 3B. E3S was docked and shown in all the above docking poses. A) Hydrogen bond interactions. B) Water mediated interactions. C) Hydrophobic interactions. D) All interactions combined (Hydrogen bond, water mediated, hydrophobic).

Figure S16: Multiple sequence alignment highlighting conserved residues and motifs.

Figure S17: Electrostatic potential surfaces along the pore of Class 1(A-C), class 2(D,E) and class 3 (F-H) OATP transporters

Figure S18: Hydrophobic surface volumes along the pore of Class 1(A-C), class 2(D,E) and class 3 (F-H) OATP transporters

Figure S19: A) Hydrophobic volumes shown in the table, B) Hydrophobic volumes shown in bars for each OATP transporter

Figure S20: A-H) Pore diameter vs X-coordinates shown for their respective OATP transporters.

Figure S21: I-K) Pore diameter vs X-coordinates shown for OATP4C1, OATP5A1 and OATP6A1.

Table S1: Model validation report from Ramachandran plots

Protein	Ramachandran Favored region residue percentage	Number of residues in disallowed region
OATP1A2	96.0%	4
OATP1B1	96.8%	1
OATP1B3	95.9%	3
OATP1C1	96.6%	5
OATP2A1	94.9%	1
OATP2B1	97.0%	2
OATP3A1	96.2%	6
OATP4A1	96.9%	1
OATP4C1	96.8%	2
OATP5A1	96.2%	3
OATP6A1	96.5%	3

Table S2: SiteMap scores for the binding site regions of OATPs. SiteMap prediction for the potential binding sites in all OATPs. The SiteScore is >0.8 for all the OATPs which depicts the druggability of the sites. The table also includes the metrics calculated by the SiteMap Volume, size, Hydrophobic, hydrophilic and donor/acceptor sites.

OATP	SiteScore	Volume (Å)	Size	Hydrophobic	Hydrophilic	Donor/Acceptor
OATP1A2	1.11	1264	359	1.51	0.72	1.01
OATP1B1	1.13	1556	539	1.50	0.81	1.36
OATP1B3	1.17	605	202	1.45	0.85	0.88
OATP1C1	1.08	1288	457	1.14	0.89	1.09
OATP2A1	1.19	549	197	1.63	0.75	1.07
OATP2B1	1.20	739	341	2.26	0.66	0.78
OATP3A1	1.23	335	162	2.57	0.66	2.59
OATP4A1	1.12	1627	586	1.26	0.93	1.62
OATP4C1	1.20	971	356	2.47	0.63	1.96
OATP5A1	1.13	1276	523	1.58	0.82	0.86
OATP6A1	1.16	760	238	1.61	0.79	1.72

Table S3: RMSD (Transmembrane helices) calculated by aligning alpha fold models and our OATP models

OATP	RMSD (Å)
OATP1A2	2,65
OATP1B1	2,72
OATP1B3	2,59
OATP1C1	2,31
OATP2A1	2,00
OATP2B1	1,79
OATP3A1	3,19
OATP4A1	1,97
OATP4C1	2,72
OATP5A1	2,37
OATP6A1	2,76

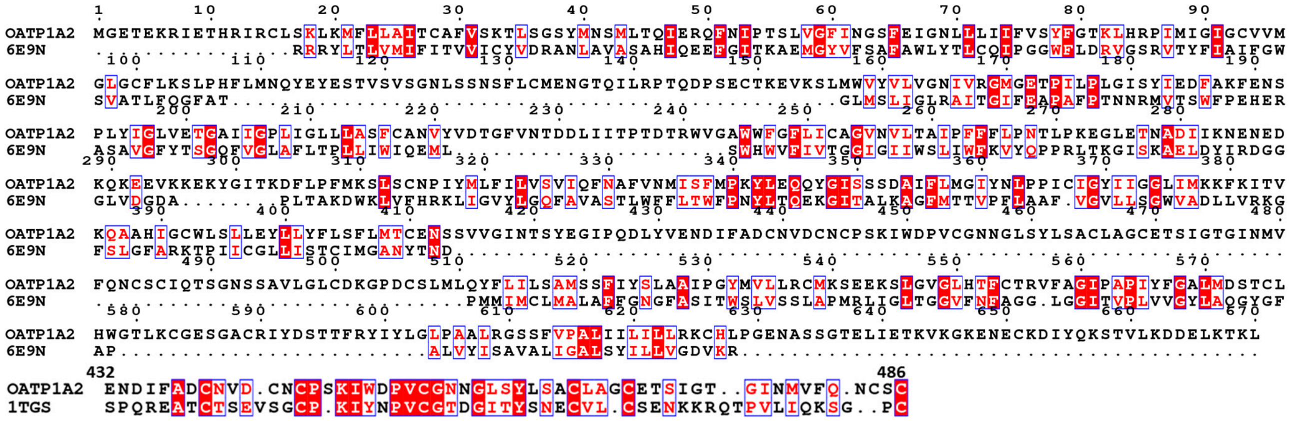

Figure S1: OATP1A2 alignment with 6E9N and 1TGS

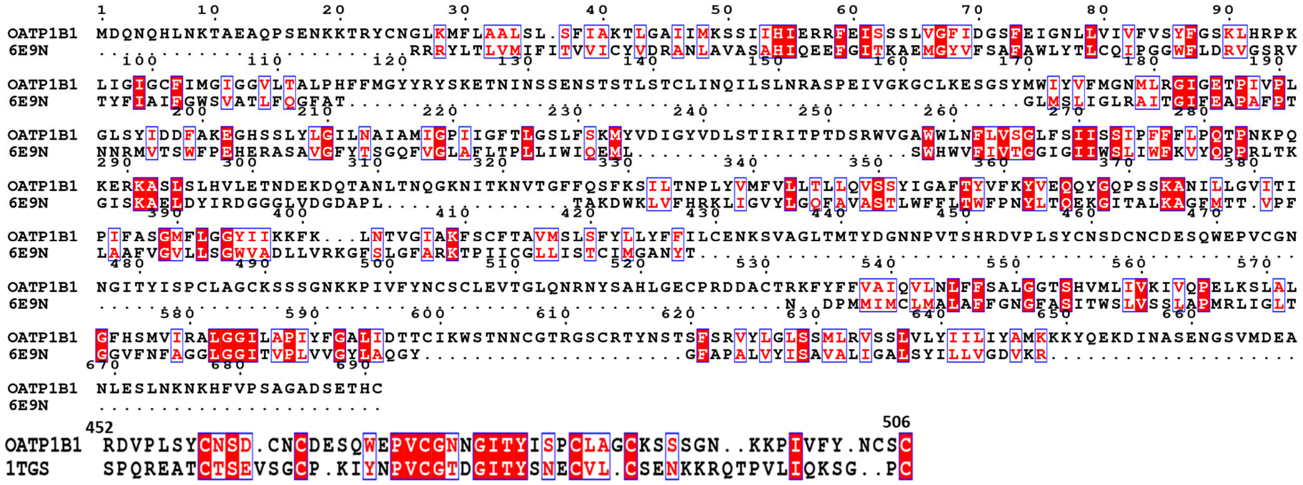

Figure S2: OATP1B1 alignment with 6E9N and 1TGS

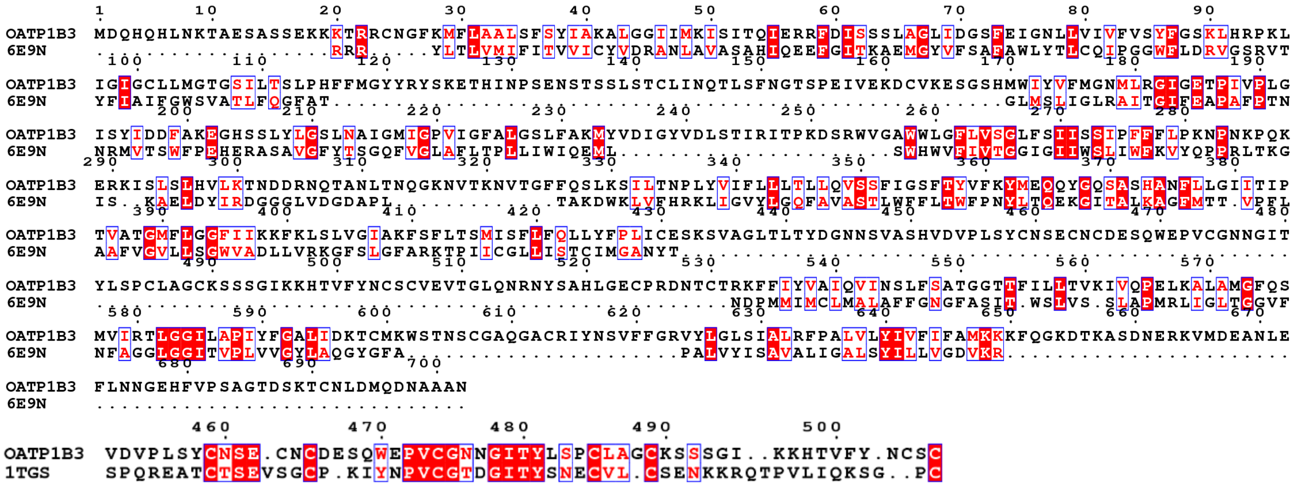

Figure S3: OATP1B3 alignment with 6E9N and 1TGS

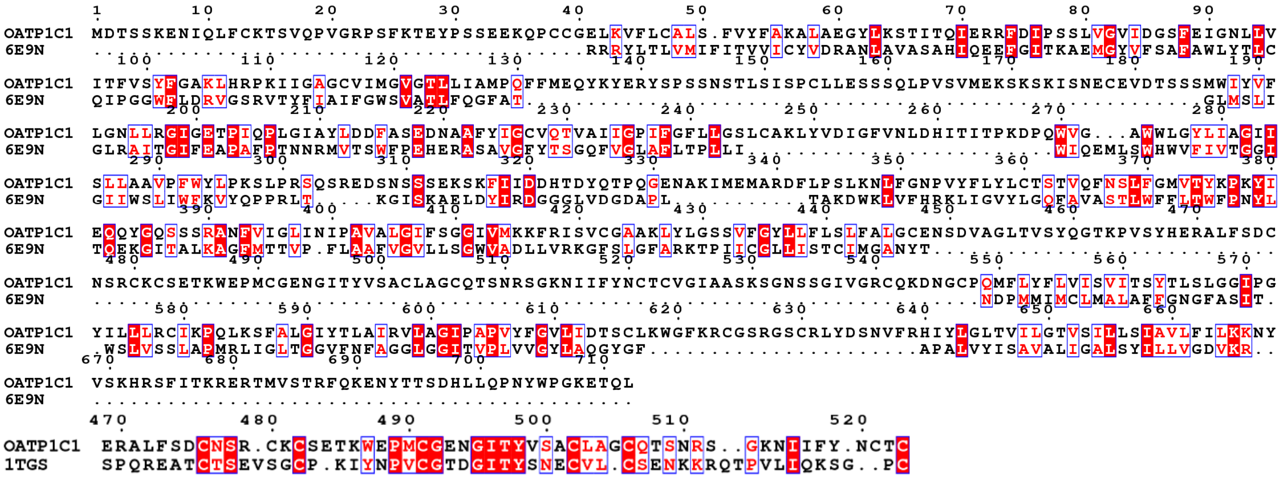

Figure S4: OATP1C1 alignment with 6E9N and 1TGS

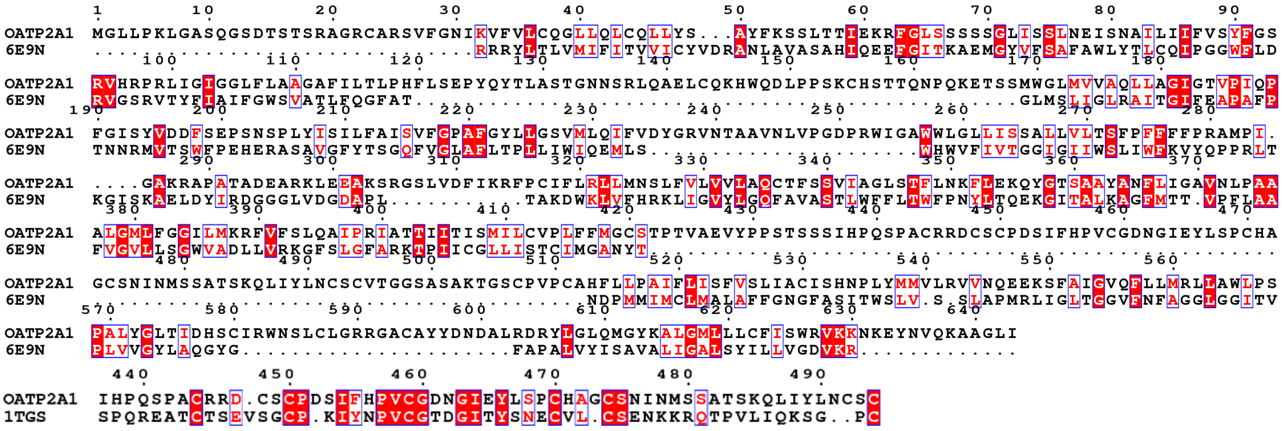

Figure S5: OATP2A1 alignment with 6E9N and 1TGS

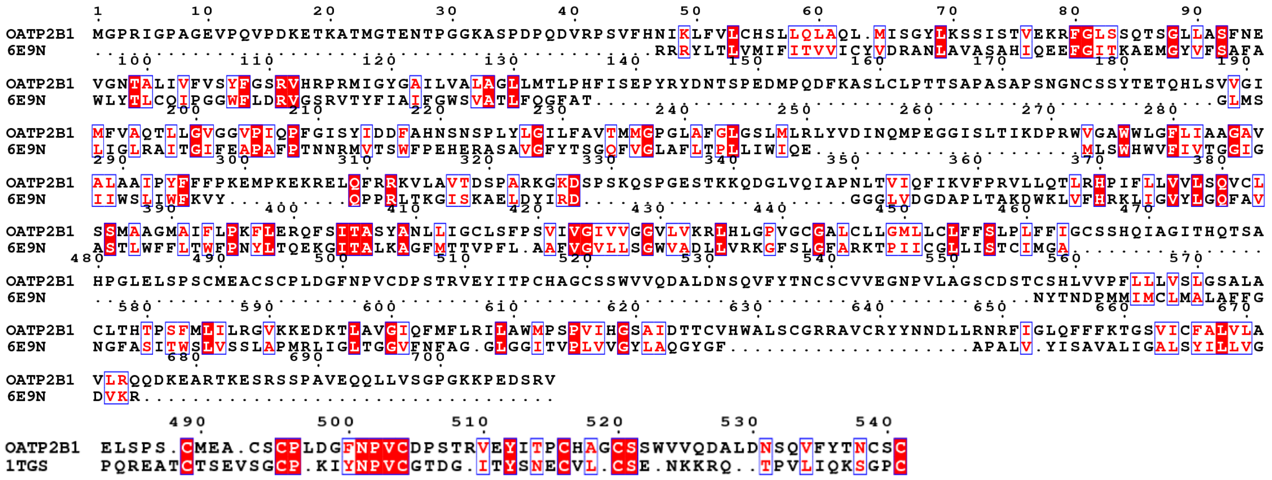

Figure S6: OATP2B1 alignment with 6E9N and 1TGS

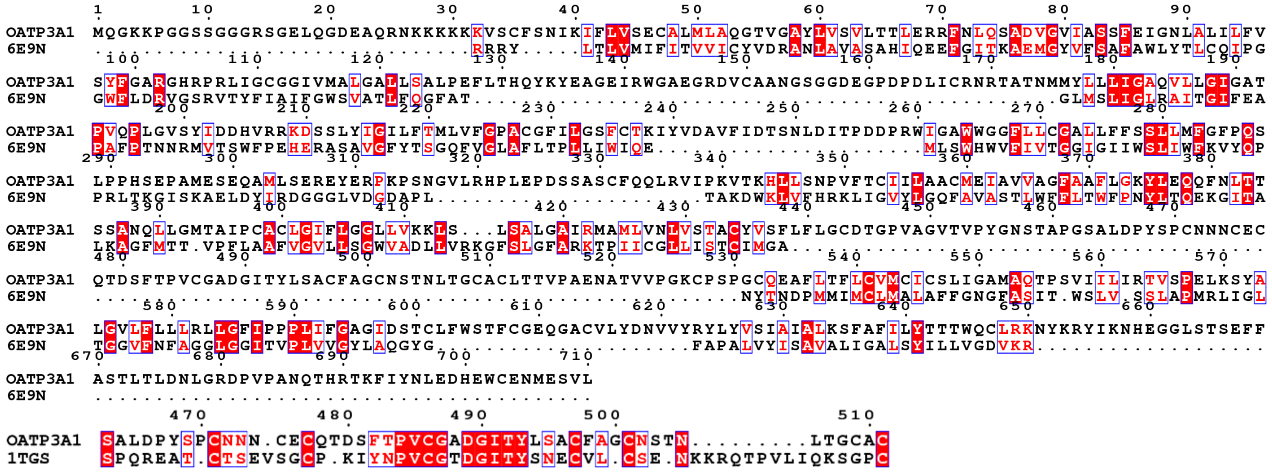

Figure S7: OATP3A1 alignment with 6E9N and 1TGS

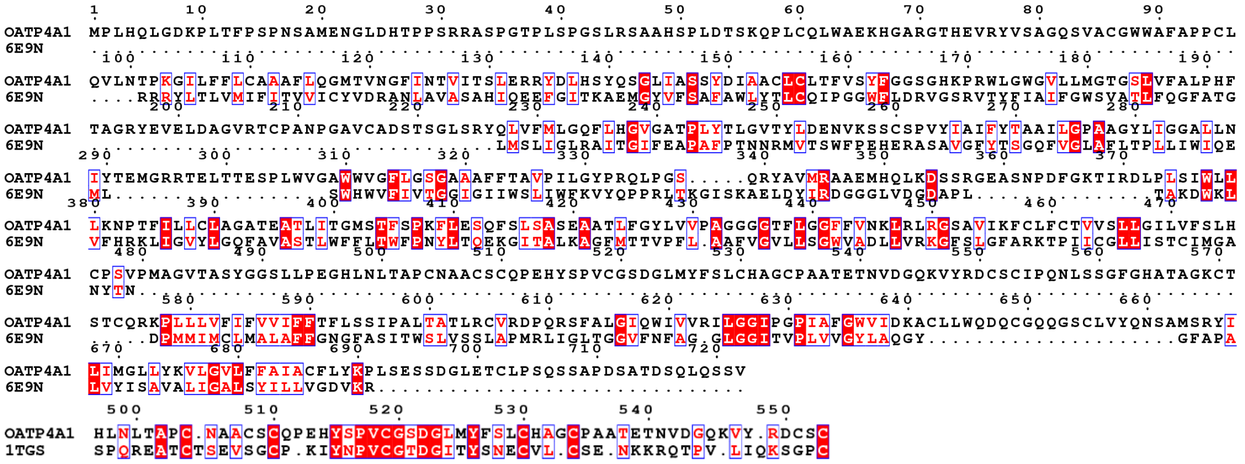

Figure S8: OATP4A1 alignment with 6E9N and 1TGS

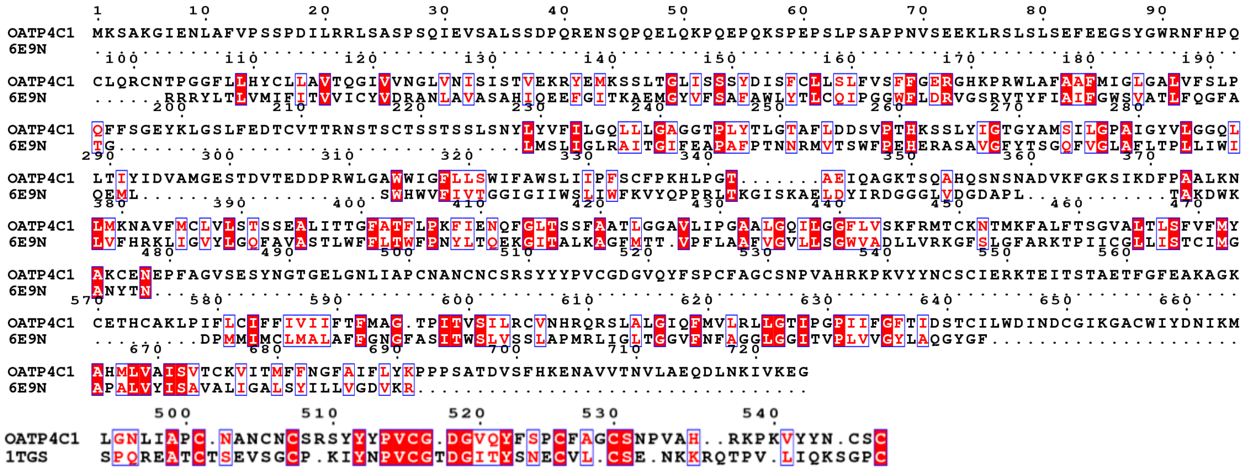

Figure S9: OATP4C1 alignment with 6E9N and 1TGS

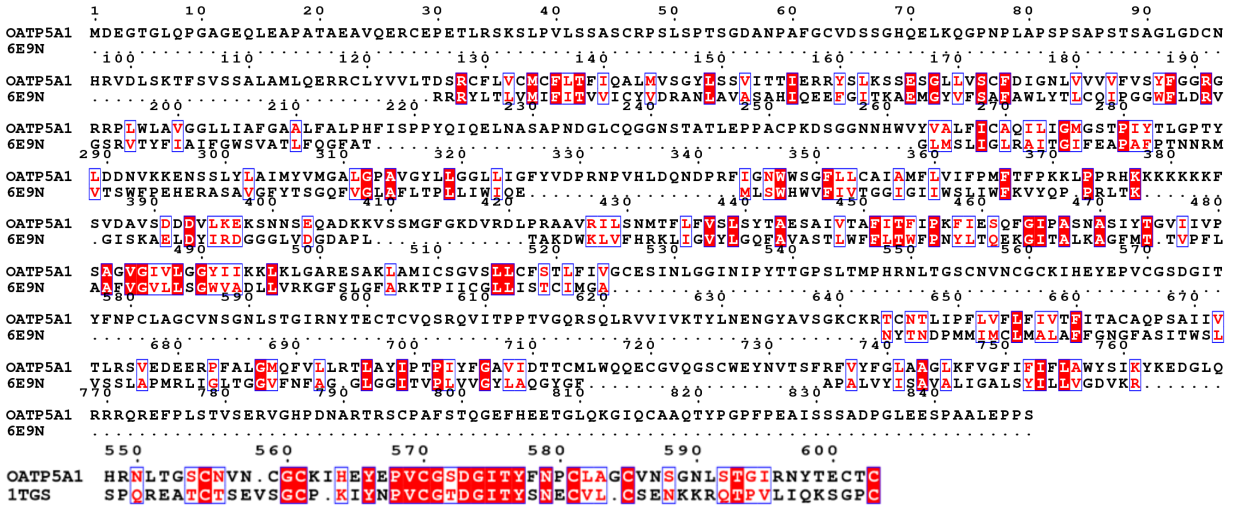

Figure S10: OATP5A1 alignment with 6E9N and 1TGS

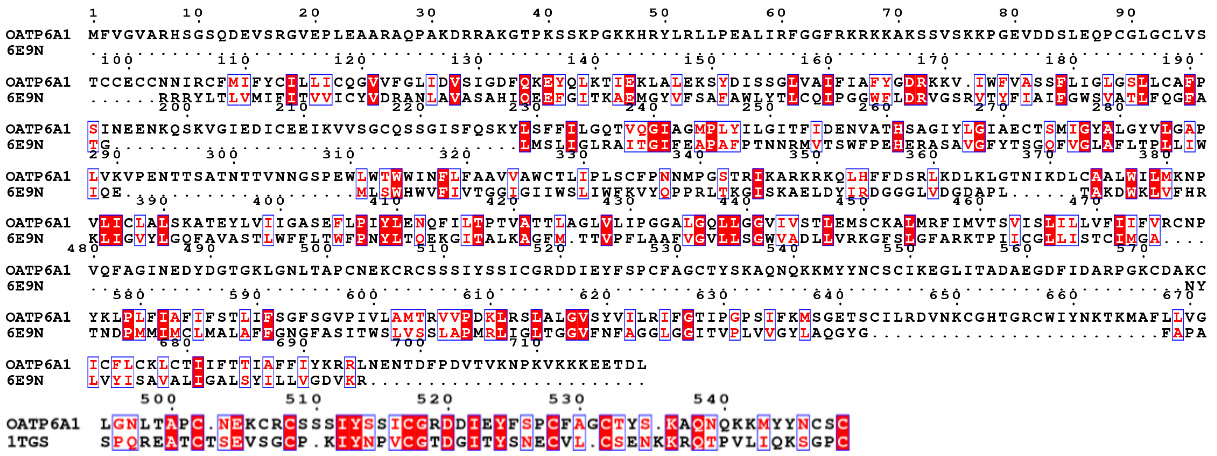

Figure S11: OATP6A1 alignment with 6E9N and 1TGS

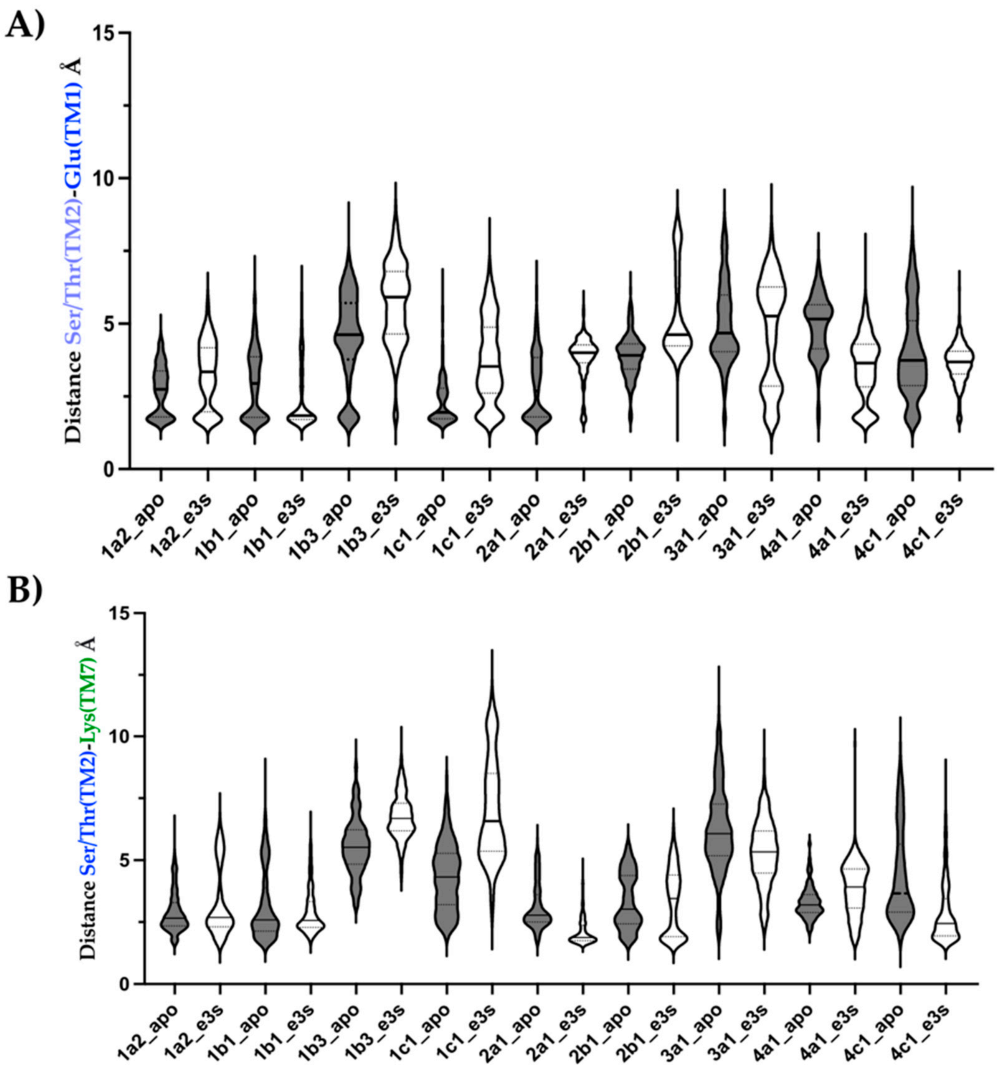

Figure S12: Distances along the molecular dynamics simulations of OATPs. A) Distance between Ser/Thr on TM2 vs Glu on TM1 observed along the simulations. B) Distance between Ser/Thr on TM2 vs Lys on TM7.

Identity	OATP1A2	OATP1B1	OATP1B3	OATP1C1	OATP2A1	OATP2B1	OATP3A1	OATP4A1	OATP4C1	OATP5A1	OATP6A1
**OATP1A2**	100	47,22	33,33	55,56	19,44	22,22	13,89	25	8,33	22,22	5,56
**OATP1B1**	47,22	100	69,44	41,67	22,22	25	25	19,44	11,11	22,22	11,11
**OATP1B3**	33,33	69,44	100	38,89	25	22,22	25	8,33	11,11	22,22	8,33
**OATP1C1**	55,56	41,67	38,89	100	22,22	27,78	22,22	22,22	11,11	25	8,33
**OATP2A1**	19,44	22,22	25	22,22	100	61,11	30,56	13,89	13,89	25	13,89
**OATP2B1**	22,22	25	22,22	27,78	61,11	100	38,89	13,89	27,78	33,33	13,89
**OATP3A1**	13,89	25	25	22,22	30,56	38,89	100	11,11	25	30,56	19,44
**OATP4A1**	25	19,44	8,33	22,22	13,89	13,89	11,11	100	47,22	33,33	27,78
**OATP4C1**	8,33	11,11	11,11	11,11	13,89	27,78	25	47,22	100	33,33	38,89
**OATP5A1**	22,22	22,22	22,22	25	25	33,33	30,56	33,33	33,33	100	19,44
**OATP6A1**	5,56	11,11	8,33	8,33	13,89	13,89	19,44	27,78	38,89	19,44	100

A. Identity matrix of binding sites of OATPs

Similarity	OATP1A2	OATP1B1	OATP1B3	OATP1C1	OATP2A1	OATP2B1	OATP3A1	OATP4A1	OATP4C1	OATP5A1	OATP6A1
**OATP1A2**	100	75	58,33	69,44	30,56	33,33	36,11	41,67	30,56	33,33	33,33
**OATP1B1**	75	100	83,33	63,89	36,11	38,89	50	47,22	36,11	41,67	36,11
**OATP1B3**	58,33	83,33	100	61,11	36,11	38,89	55,56	41,67	44,44	44,44	41,67
**OATP1C1**	69,44	63,89	61,11	100	38,89	47,22	50	50	41,67	50	41,67
**OATP2A1**	30,56	36,11	36,11	38,89	100	83,33	50	33,33	38,89	47,22	38,89
**OATP2B1**	33,33	38,89	38,89	47,22	83,33	100	63,89	36,11	50	52,78	41,67
**OATP3A1**	36,11	50	55,56	50	50	63,89	100	47,22	58,33	63,89	52,78
**OATP4A1**	41,67	47,22	41,67	50	33,33	36,11	47,22	100	55,56	55,56	44,44
**OATP4C1**	30,56	36,11	44,44	41,67	38,89	50	58,33	55,56	100	55,56	55,56
**OATP5A1**	33,33	41,67	44,44	50	47,22	52,78	63,89	55,56	55,56	100	41,67
**OATP6A1**	33,33	36,11	41,67	41,67	38,89	41,67	52,78	44,44	55,56	41,67	100

B. Similarity matrix of binding sites of OATPs

Figure S13: Identity and similarity matrices of binding sites of OATPs: A) Identity matrix of binding sites of OATPs. B) Similarity matrix of binding sites of OATPs.

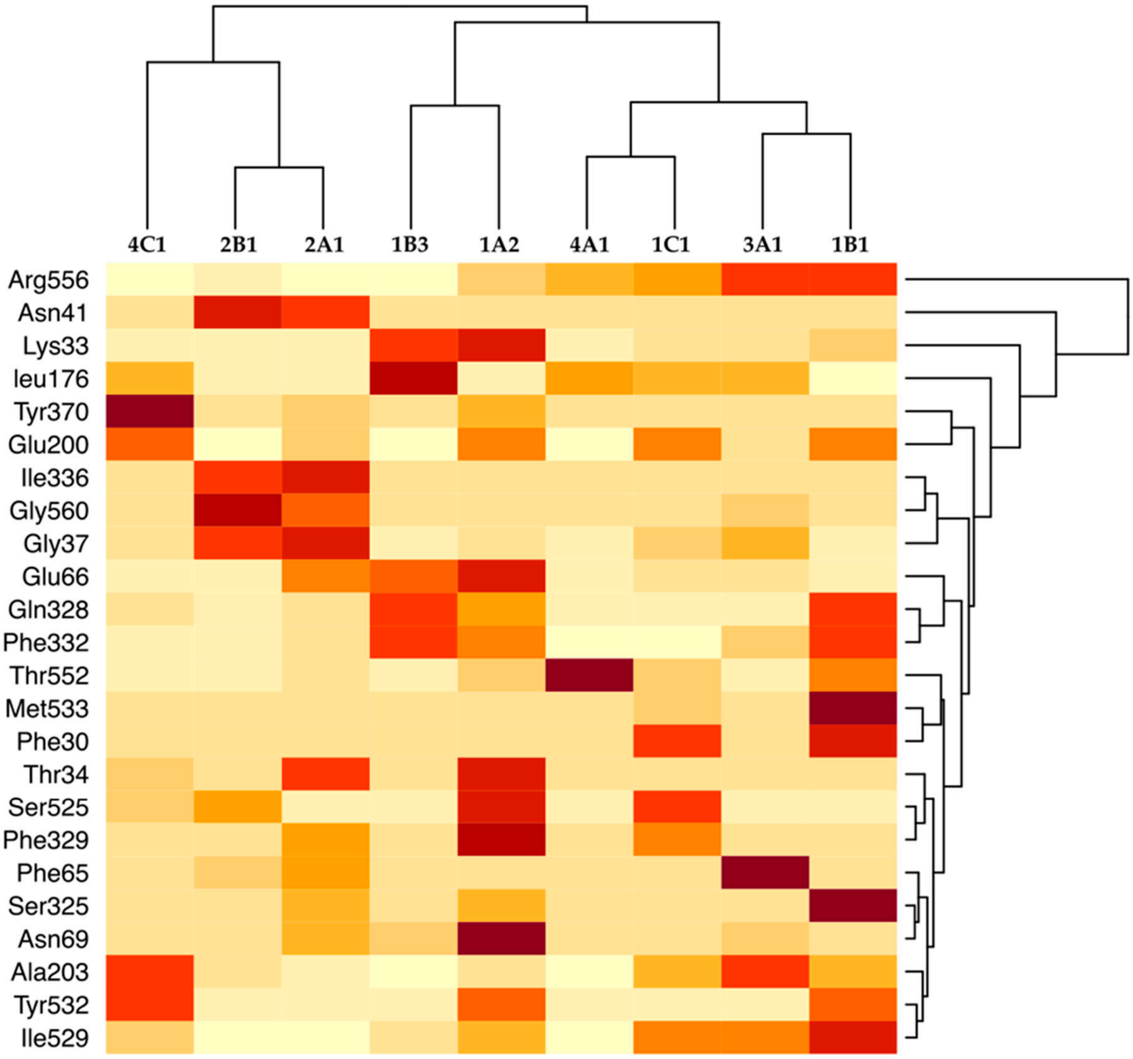

Figure S14: Classification of OATPs based on the interactions observed in the molecular dynamics simulations.

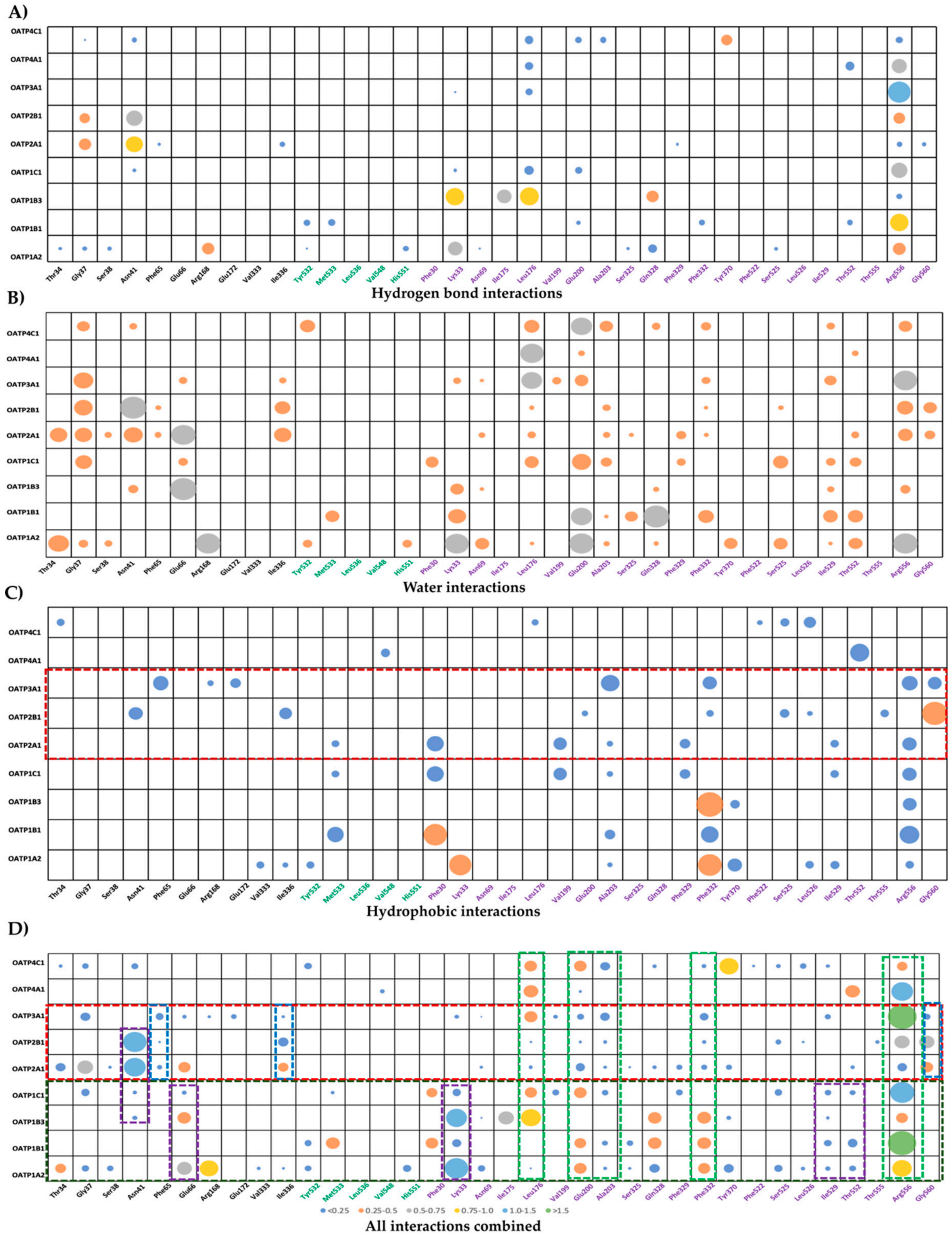

Figure S15: Frequency of protein-ligand interactions along the molecular dynamics simulation trajectories are shown in bubble graphs, the residues are named according to OATP1A2, and the corresponding aligned residues could be referred from Figure 3B. E3S was docked and shown in all the above docking poses. A) Hydrogen bond interactions. B) Water mediated interactions. C) Hydrophobic interactions. D) All interactions combined (Hydrogen bond, water mediated, hydrophobic).

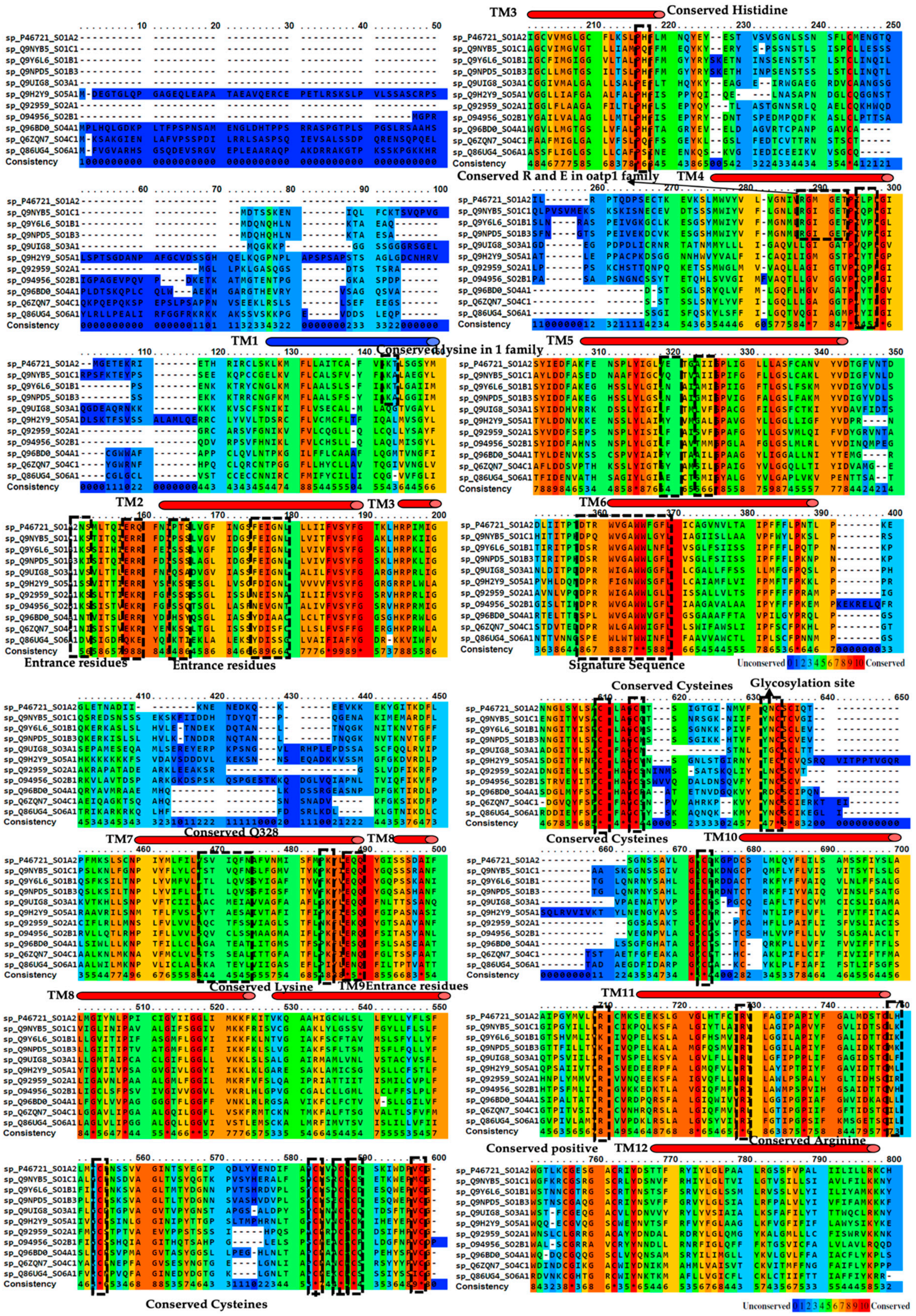

Figure S16: Multiple sequence alignment highlighting conserved residues and motifs

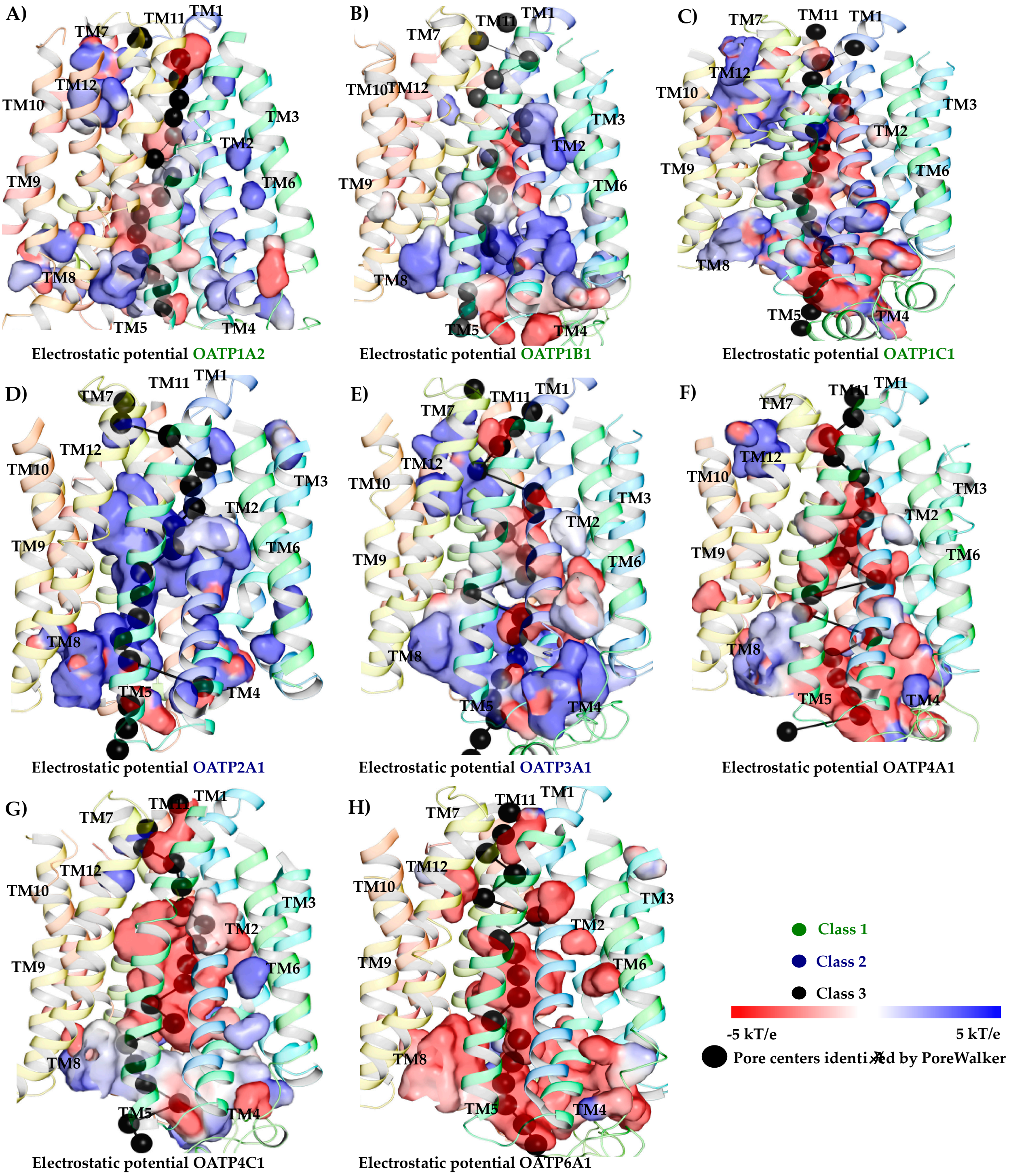

Figure S17: Electrostatic potential surfaces along the pore of Class 1(A-C), class 2(D,E) and class 3 (F-H) OATP transporters

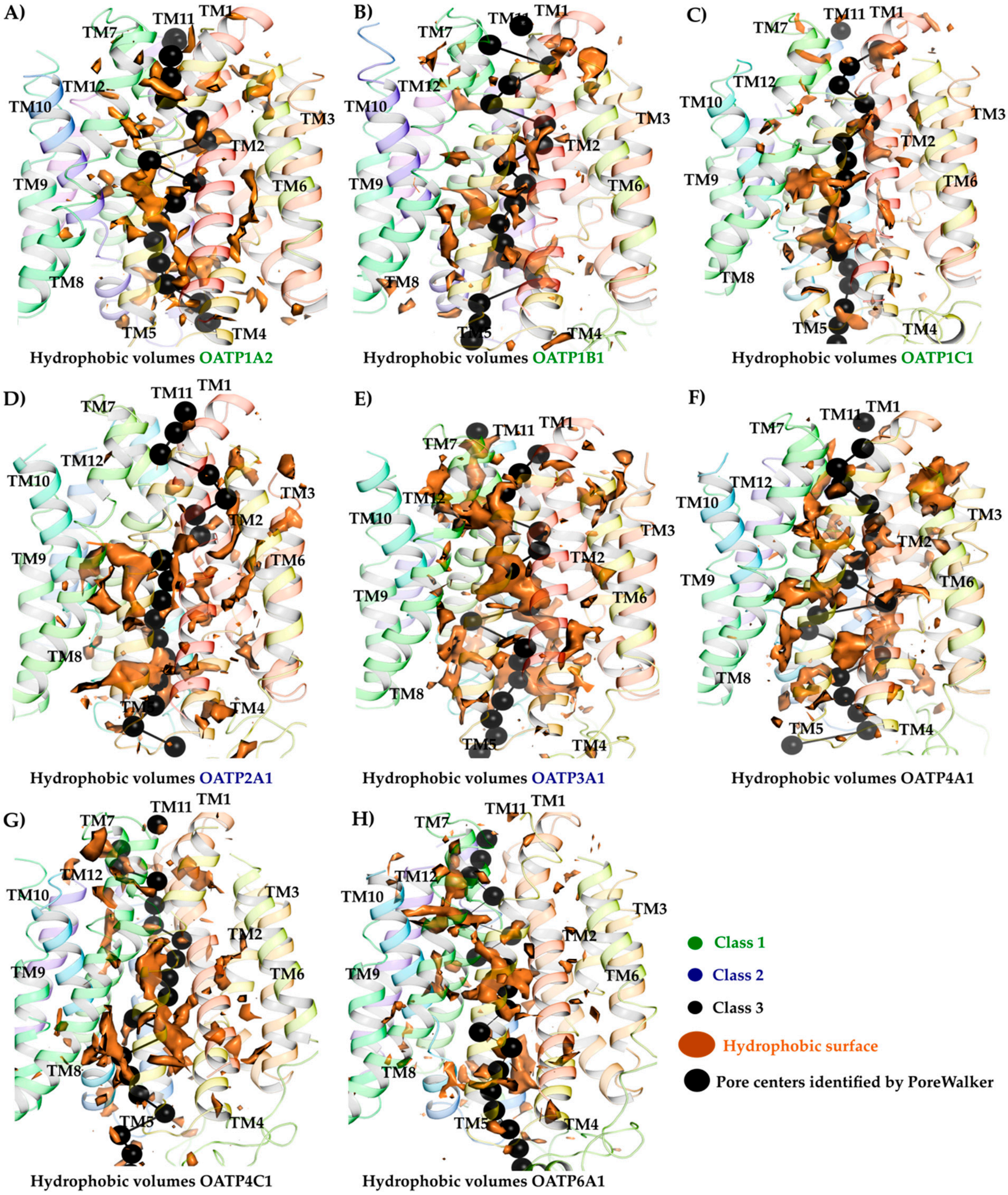

Figure S18: Hydrophobic surface volumes along the pore of Class 1(A-C), class 2(D,E) and class 3 (F-H) OATP transporters

A)

Class	Transporter	Hydrophobic_Volume
class1	OATP1A2	796
class1	OATP1B1	759
class1	OATP1B3	1088
class1	OATP1C1	867
class2	OATP2A1	1125
class2	OATP2B1	1032
class2	OATP3A1	1527
class3	OATP4A1	1240
class3	OATP4C1	1161
class3	OATP5A1	1012
class3	OATP6A1	1008

B)

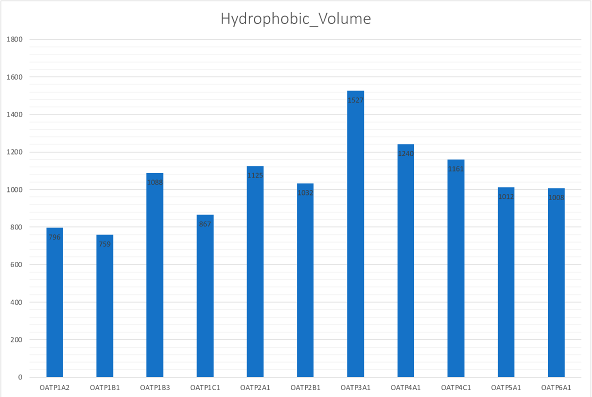

or

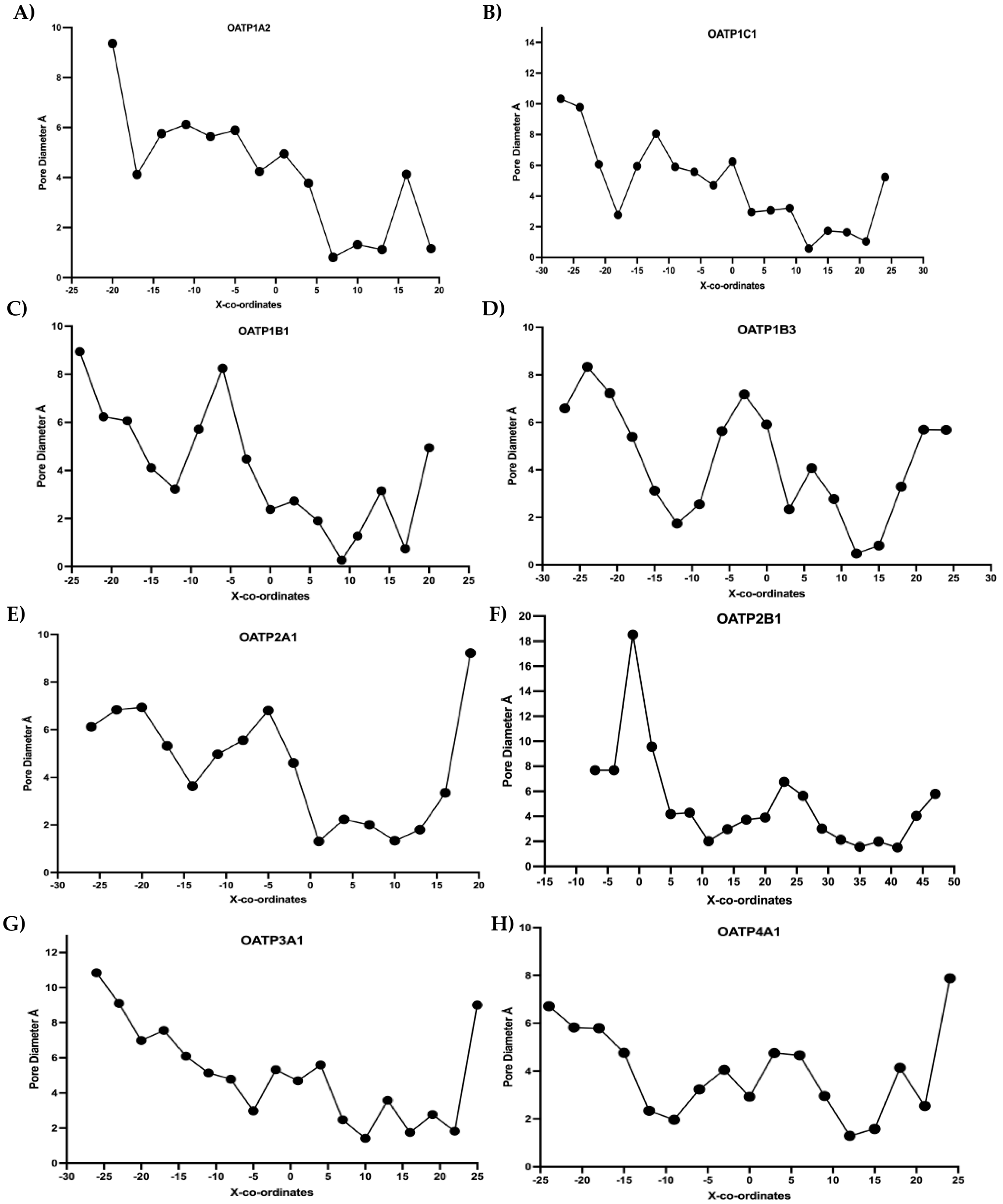

Figure S20: A-H) Pore diameter vs X-coordinates shown for their respective OATP transporters.

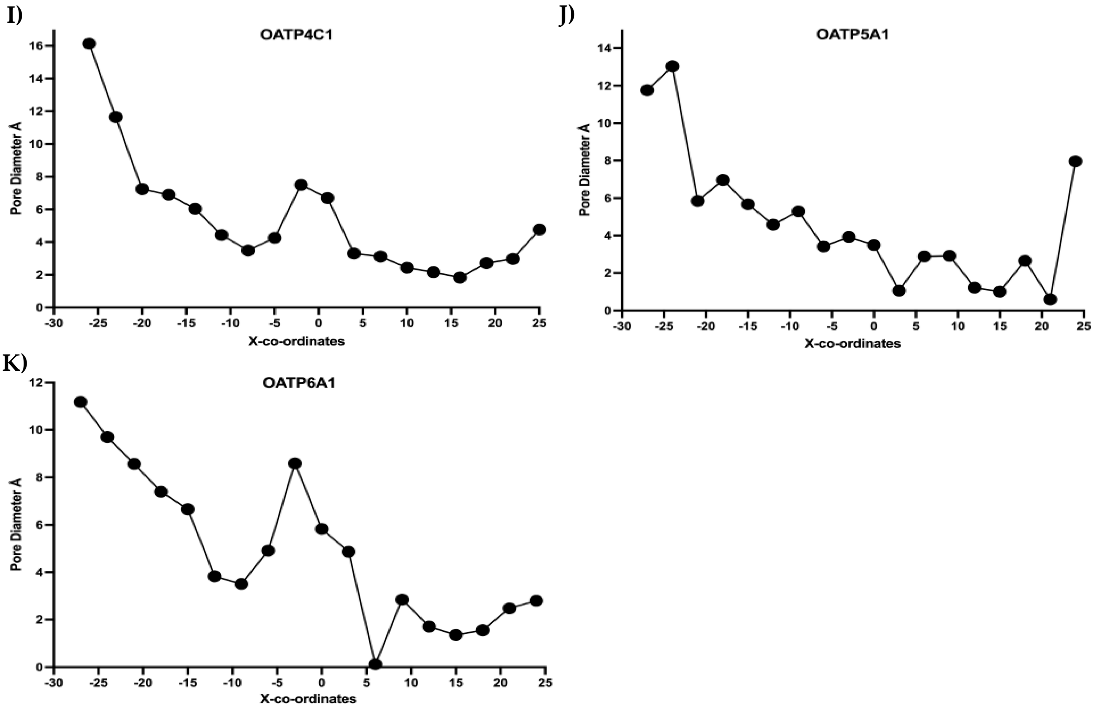

Figure S21: I-K) Pore diameter vs X-coordinates shown for OATP4C1, OATP5A1 and OATP6A1.
